# Sad Case of Death by Careless Renewal of a Prescription

**Published:** 1869-01

**Authors:** 


SAD CASE OF DEATH BY CARELESS RENEWAL OF A PRESCRIPTION.—Dr. Philip DeYoung, an experienced physician of Philadelphia, recently prescribed a cathartic pill, composed in part of three grains of assafœtida, for his sister, Mrs. Hecht. The pills were renewed several times, but at last, by some terrible ignorance or carelessness, three grains of *atropine* were substituted for the assafœtida, of course with a speedy and fatal result.

